# A self-controlled, cross-over study of intensive insulin treatment with needle-based injection versus needle-free injection in hospitalized patients with type 2 diabetes

**DOI:** 10.3389/fendo.2023.1162176

**Published:** 2023-07-12

**Authors:** Quanying Wu, Mingqun Deng, Weihao Wang, Shuyi Yu, Miao Wang, Chao Sun, Qi Pan, Lixin Guo

**Affiliations:** ^1^ Nursing Department, Beijing Hospital, National Center of Gerontology, Institute of Geriatric Medicine, Chinese Academy of Medical Sciences, Beijing, China; ^2^ Department of Endocrinology, Beijing Hospital, National Center of Gerontology, Institute of Geriatric Medicine, Chinese Academy of Medical Sciences, Beijing, China

**Keywords:** needle-free injection, diabetes, intensive insulin therapy, in hospital, cross-over study

## Abstract

**Background and Aims:**

Needle injection and needle-free injection were proven effective in improving glycated hemoglobin (HbA1c) in type 2 diabetes mellitus (T2DM) patients. However, it is unclear if needle-free and needle injections of insulin during intensive insulin therapy in hospitalized patients provide similar efficacy and safety benefits.

**Methods:**

A self-controlled cross-over study was conducted on 62 patients with T2DM who received intensive long-acting and short-acting insulin injections with or without needles. The 7-point blood glucose test was performed on the 6th day after insulin administration and the injection method switched on the 7th day of hospitalization. The difference was compared in 7-point blood glucose levels.

**Results:**

The blood glucose levels at fasting (mean difference=-1.09 ± 2.38mmol/L, 95% CI, -1.69 to -0.48, p=0.0007) and post-breakfast (-1.14 ± 3.02mmol/L, 95%CI, -1.91 to -0.37, p=0.004) were better when patients were receiving needle-free injections compared to when receiving a needle injection. Indeed, daily blood glucose fluctuation, which presented as the area under the curve of glycemia, was decreased in needle-free injection periods (-0.3.48 ± 9.64, 95%CI, -5.95 to -1.01, p=0.0065). There was no significant difference in the dose of long-acting insulin between the two injection methods (-0.32 ± 2.69, 95%CI, -0.99 to 0.37, p>0.05). The dose of fast-acting insulin during the needle-free period was lower than that of when patients received needle injections (-1.66 ± 6.45, 95%CI, -3.29 to -0.025, p<0.05). There was no significant difference in satisfaction between the two regimens (-0.59 ± 1.55,95%CI, -0.938 to 0.509, p=0.557), but there was a significant difference in pain experience, favoring needle-free injections (p < 0.001).

**Conclusion:**

Glycemia was better controlled by needle-free insulin injections in hospitalized T2DM patients subjected to intensive glycemic control. These patients also experienced less pain than when insulin was injected with a needle.

## Introduction

Insulin plays an important role in the treatment of diabetes mellitus. With the increasing prevalence of diabetes mellitus in China, an increasing number of patients develop this disease. A cross-sectional 3B study in China showed that 35.7% of T2DM individuals are treated with insulin injections (1). The 2021 Global Diabetes map shown by the International Diabetes Federation (IDF) reiterates that China ranks first in the number of people with diabetes and third in the cost of diabetes-related treatment (2). Regular insulin injections can control the progression of diabetes and prevent the occurrence of complications (3–6). However, injection with a needle are associated with pain and fear in these patients, while repeated use of needles leads to subcutaneous fat hyperplasia, bleeding, and other conditions that ultimately result in treatment interruption, blood glucose control, and quality of life (7, 8).

The needle-free syringe is a new type of insulin delivery device which has received extensive attention in recent years. Needle-free injections, also known as jet injections, are the application of the principle of pressure jets to complete the subcutaneous injection of liquid medicine (9). The needle-free syringe internal pressure device can produce pressure and the liquid instantly penetrates the human skin. The liquid medicine is then diffused under the skin and exerts its effect quickly (10). The 2016 edition of the Chinese technical guidelines for the injection of diabetes drugs recommended the use of a needle-free syringe for insulin injection as one of the most used insulin injection devices in clinical practice (11). Many studies have confirmed that, compared with needle injections, insulin is more quickly absorbed when administrated by needle-free systems, which is similar to the physiological insulin secretion pattern. Needle-free injections are also associated with a better experience and blood glucose control in diabetic patients (12). Needle-free injections can effectively reduce injective pain and fear, and improve patient compliance. Thus, this injection regimen is becoming widely used in clinical practice.

At present, most studies have been performed based on the conversion from needle to needle-free injection. No cross-over study was carried out to compare the effectiveness and satisfaction between these injection regimens. Therefore, the aim of this study was to compare the impact of needle-free and needle injections on the clinical outcomes, and the injection experience in inpatients with type 2 diabetes mellitus (T2DM) receiving intensive treatment.

## Materials and methods

### Data sources

Clinical data were collected by convenient sampling according to the time of admission. From November 2018 to June 2020, 64 T2DM patients who received intensive treatment of long- and short-acting insulins were selected in the department of endocrinology at Beijing Hospital. Two participants in Group A dropped out during the treatment period and 62 completed the study. A total of 25 patients in Group A were given a needle-free insulin injection for 6 days and then switched to needle injections for 7 days. Meanwhile, 37 patients in Group B were given needle injections for 6 days, and then switched to needle-free injections for 7 days.

#### Inclusion criteria

1. patients who met the 1999 WHO diagnostic criteria for diabetes; 2. patients who received long-acting insulin injections > 4IU (no restrictions on combined oral medication and short-acting/rapid-acting insulin therapy); 3. patients with full cognitive and behavioral ability; 4. patients who signed informed consent and voluntary use of needle-free syringes.

#### Exclusion criteria

1. patients who refused to use needle-free syringes; 2. patients with mental illnesses; 3. patients with disabilities such as blindness and deafness; 4. patients who used insulin for the first time; 5. patients who used insulin pumps and disposable syringes for insulin injection; 6. patients who used needle-free insulin injection for a long time; 7. patients who received <4IU long-acting insulin; 8. patients who changed the injection dose due to glucose fluctuations after changing to needle injection, and patients with acute complications such as acute infections, ketoacidosis, and severe water and electrolyte disturbances; 9. patients who changed the dose and type of oral medications during the study period.

This study was approved by the hospital ethics committee (2020BJYYE c-026-01), and all subjects signed the informed consent form.

### Study design

**Figure 1:**
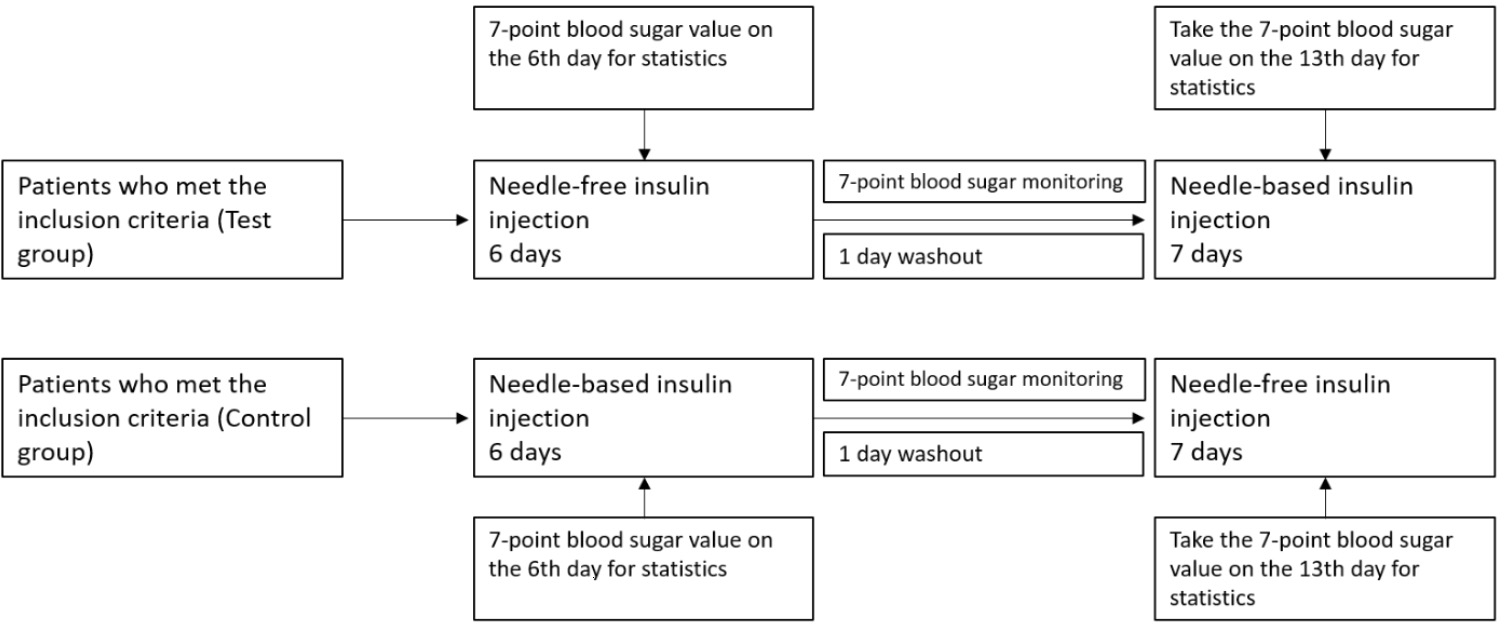
Clinical study design. Here it is shown the clinical design of the test group and the control group. Insulin with a needle injection system or needle-free injections was used for intensive treatment for 6 days. On the 6^th^ day of treatment, blood glucose was measured at 7 different time points and compared between groups. The first day after the injection mode was changed was the washing-out period.

This was a self-control study. After intensive treatment, T2DM hospitalized patients received unified health education guidance from diabetes specialist nurses in the department of endocrinology. Subjects first took a needleless syringe QS-P (Beijing Kuaishuer Medical Technology Co., Ltd.), or a needle insulin pen to inject long-acting insulin (Lantus pre-filled Solotar, 300IU/3mL, Sanofi-Aventis; or Insulin Degludec injection 300IU/3mL, Novo Nordisk (China) Pharmaceutical Co., Ltd.), and fast-acting insulin (Insulin Aspartame Injection 300IU/3mL, Novo Nordisk (China) Pharmaceutical Co., Ltd.). After 6 days, the injection method changed, with a one-day drug washout period in between (see **Figure 1**). The fast glucose meter was used to measure blood glucose levels in the fingertips 7 times a day (time points before and after three meals and before going to bed).

To avoid the influence of injection technique and injection method on the results, both injection methods were performed by nurses who had worked in the department of endocrinology for more than 1 year. Nurses were given injection training before applying them. The formulation of the operational standards referred to the 2016 edition of “China Technical Guidelines for Diabetes Drug Injection” (11) and the “Work Guideline for needle-free Insulin injection to diabetics” (13). Glycemia was evaluated on the 6th day after the start of the first injection period (eluting the effect of long-acting insulin).

### Main outcomes

The primary endpoint was glucose control in both groups. Indicators of glucose control included seven-point glucose levels, intraday glucose fluctuations, and the daily dose of long-acting and fasting-acting insulin for the two insulin injection methods on the 6th-day using such method. Seven-point glucose testing (fasting, after breakfast, before lunch, after lunch, before dinner, after dinner) is required in patients with type 2 diabetes who require intensive treatment. The seven-point glucose value and the daily dose of long-acting and rapid-acting insulin was recorded on the 6^th^ day after using each injection method.

Secondary outcomes included the overall satisfaction of patients with each drug delivery device and the pain level of patients. The overall satisfaction questionnaire adopted the Likert five-point scoring method, and the scores from high to low were: very satisfied (9-10 points), satisfied (8-9 points), average (6-7 points), dissatisfied (4-5 points), very dissatisfied (1-3 points). A visual analog scale (Vas) was used for evaluation, with 0 scores indicating no pain and 10 scores indicating maximum pain. A score from 0 to 3 was classified as mild pain, a score from 4 to 6 was classified as moderate pain, and a score from 7 to 10 was classified as severe pain. In this study, the patient’s experience of pain was evaluated using a pain questionnaire after two successful insulin injections in the abdomen.

### Statistical method

SPSS 20.0 and Graphpad Prism 9 were used for statistical analyses and chart plotting. The normality of continuous variables was assessed by the Shapiro-Wilk test. Quantitative data are described by means ± standard deviation ( $\bar{x}$ ± SD) when normally distributed. Non-normally distributed quantitative data are expressed as medians (interquartile range). Categorical variable data are expressed as percentages (rates).

All data showed normal distribution evidenced by the 1-sample Kolmogorov-Smirnov test using the SPSS software. One-way ANOVA and Student’s t-test were performed to compare values between the 2 groups. Bonferroni’s method was used to adjust the p-value to accommodate multiple tests if multiple comparisons were not independent of each other. The significance level was set to 0.05 for a 2-side comparison. The significance level was set to 0.007 for a single-point glucose comparison between the two groups. Intraday glucose fluctuations were compared using paired t-test (for quantitative data with normal distribution), or Wilcoxon signed-rank test (for quantitative data that did not follow a normal distribution as well as for categorical variables).

Blood glucose fluctuation was compared using the area under the curve (AUC) at 7 different time points in a day as the standard between needle injection and non-needle injection. Blood glucose levels were plotted over time after subcutaneous insulin administration in each subject. The AUC was calculated by the trapezoid method. P < 0.05 indicated that the difference was statistically significant.

## Results

A total of 62 participants were enrolled in this study. The characteristics of the participants are reported in **Table 1**. Group A included 13 men and 12 women with an average age of 60.40 years old. Group B included 22 men and 15 women with an average age of 67 years old. The mean baseline glycated hemoglobin in group A and group B were 9.98 ± 2.03 and 8.79 ± 1.44, respectively. The mean baseline BMI in group A and group B were 25.33 ± 3.55 and 24.68 ± 4.00kg/m^2^, respectively.

**Table 1:** Baseline characteristics of two groups (including sex, diabetes complications, and comorbidities).

Item		Test group (N = 25)	Control group (N = 37)	Statistical value	*P* value
Age [years, case (%)]	<60	11 (44.0)	9 (24.3)	2.841^1)^	0.242
	60~74	10 (40.0)	18 (48.6)		
	≥75	4 (16.0)	10 (27.0)		
Gender	Male	21 (58.3)	14 (53.8)	0.124^1)^	0.725
	Female	15 (41.7)	12 (46.2)		
Glycated hemoglobin [%, case (%)]	6~7	1 (4.0)	5 (13.5)	3.970^1)^	0.272
	7~8	2 (8.0)	8 (21.6)		
	8~9	8 (32.0)	8 (21.6)		
	≥9	14 (56.0)	16 (43.2)		
BMI (kg/m^2^, ± s)		25.33 ± 3.55	24.68 ± 4.00	-0.662^2)^	0.511
Fasting c-peptide [pmol/l, M (Q1, Q3)]		340.20 (166.75, 662.55)	277.90 (135.70, 375.00)	-1.399^3)^	0.162
2h C-peptide [pmol/l, M (Q1, Q3)]		891.90 (303.45, 1263.85)	666.60 (376.45, 943.90)	-0.782^3)^	0.434
Insulin [unit, M (Q1, Q3)]		21.90 (10.35,31.12)	22.70 (11.60,62.50)	-1.170^3)^	0.242
2h Insulin [unit, M (Q1, Q3)]		57.26 (28.00, 60.55)	65.00 (49.05, 99.05)	-1.563^3)^	0.118
Creatinine (μmol/l, ± s)		62.05 ± 23.44	70.22 ± 20.64	1.447^2)^	0.153
Alanine aminotransferase		21.14 ± 8.98	19.43 ± 16.14	-0.481^2)^	0.632
Triglyceride (mmol L, ± s)		1.35 (0.83,2.19)	1.19 (0.82,1.67))	-1.005^3)^	0.315
High-density lipoprotein (mmol L, ± s)		1.07 ± 0.24	1.16 ± 0.35	1.021^2)^	0.311

1) χ^2^ value; 2) t value;3) Z value. Quantitative data are described by means ± standard deviation ( $\bar{x}$ ± SD) when normally distributed. Non-normally distributed quantitative data are expressed as medians (interquartile range). Categorical variable data are expressed as percentages (rates).

**Figure 2:**
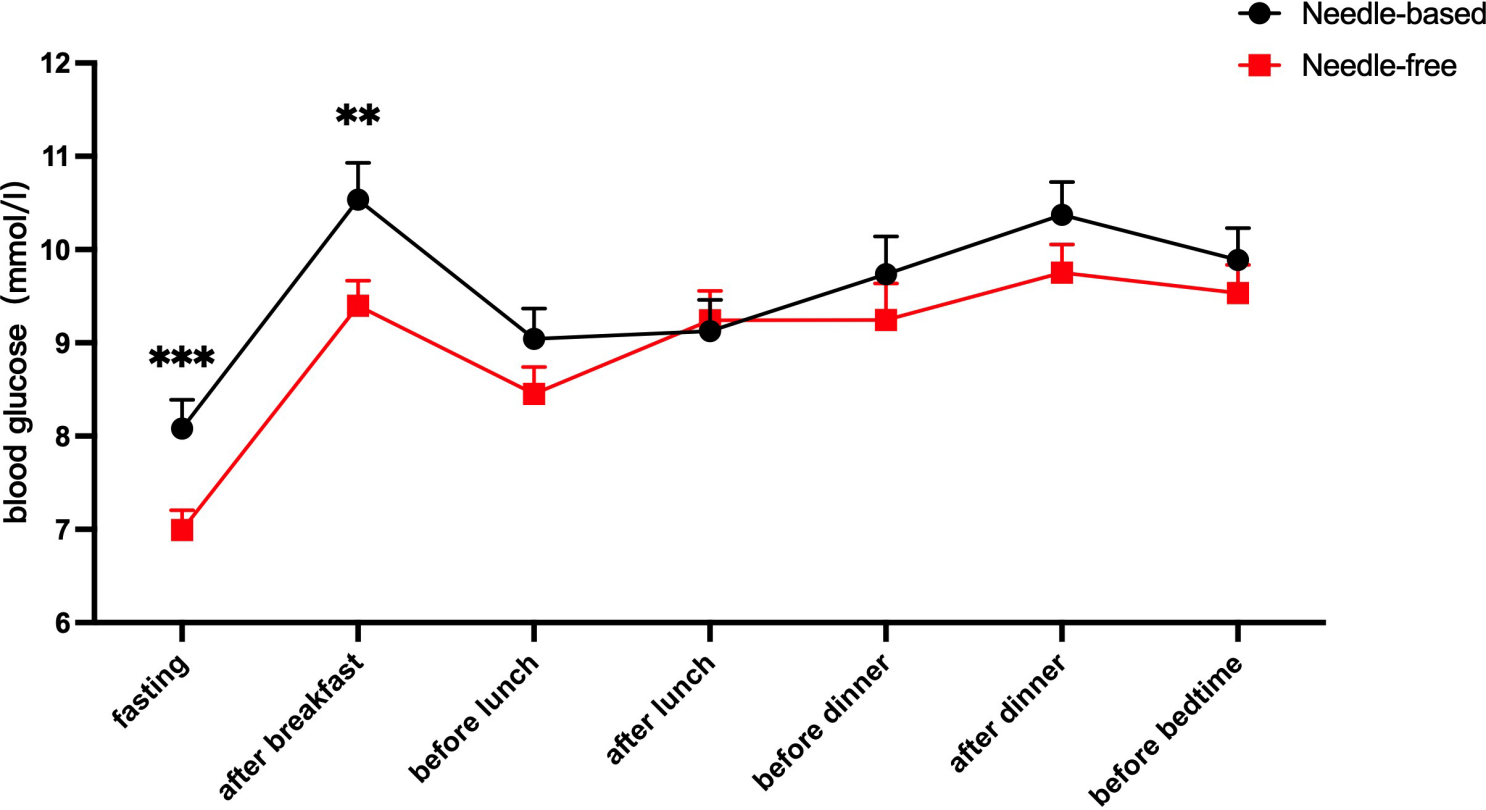
Comparison of blood glucose levels after using the two injection devices successively. The abscissa axis shows the patient’s fasting, after breakfast, before lunch, after lunch, before dinner, after dinner, and before going to bed. The ordinate is the blood glucose level (mmol/l). The red line represents the average blood glucose level at seven different time points when needle-free insulin was used, and the black line represents the average blood glucose level at the same time points when needle insulin was used. The results show that the level of blood glucose control after fasting and breakfast was improved when using a needle-free system, when compared to a needle one. ***p<0.001; **p<0.005.

**Figure 3:**
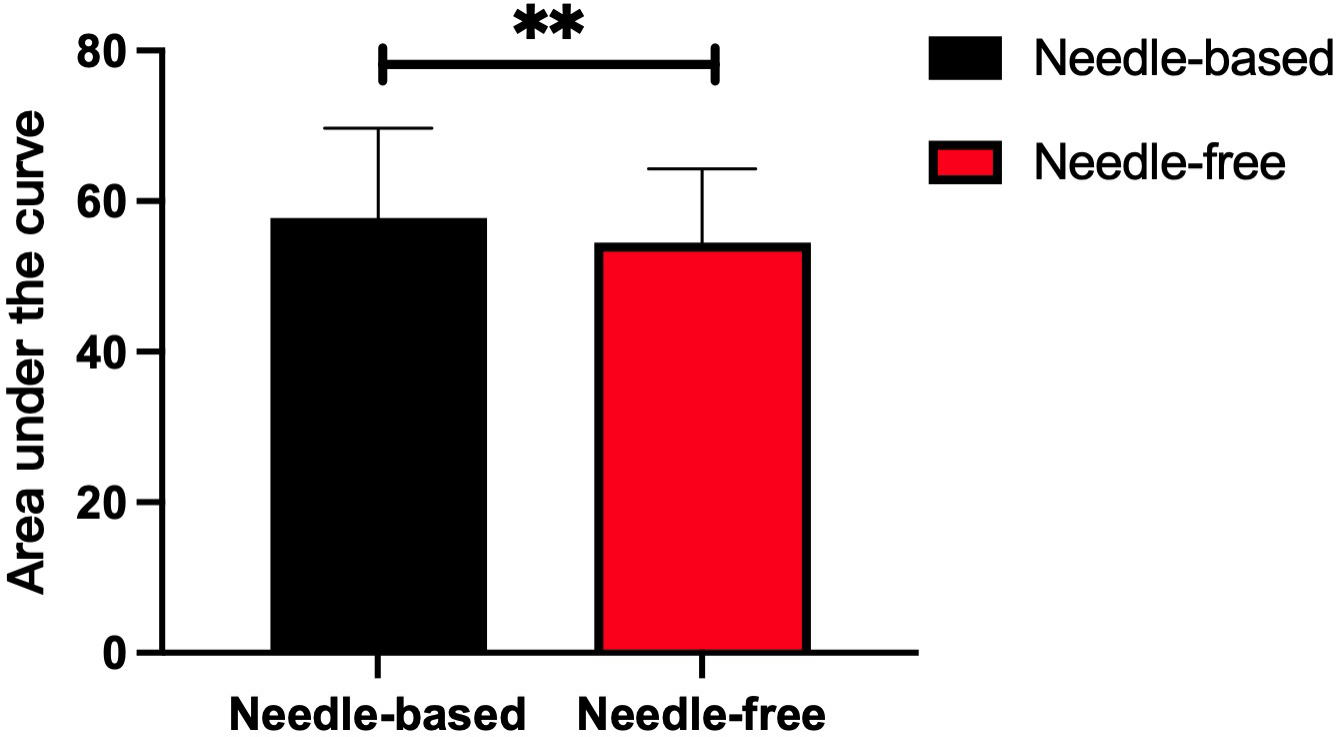
Comparison of the area under the curve after insulin injection using the two injection devices successively. The abscissa represents the two insulin injection methods, the ordinate represents the area under of the 7-point blood glucose level curve. The red box represents the area under the curve after needle-free insulin injections, and the black box represents the area under the blood glucose level curve after needle-free insulin injections. **p<0.005.

After needle-free insulin injections, the blood glucose levels were better controlled at fasting and after breakfast (p < 0.05). There was no significant difference in the control of blood glucose levels at other time points, as shown in **Figure 2**. Meanwhile, glycemia fluctuation was lower when patients received needle-free injections than when they used a needle injection device. That is, the AUC for blood glucose levels was decreased in needle-free insulin injection groups than that of groups using the needle system (p < 0.05), as illustrated in **Figure 3**.

**Figure 4:**
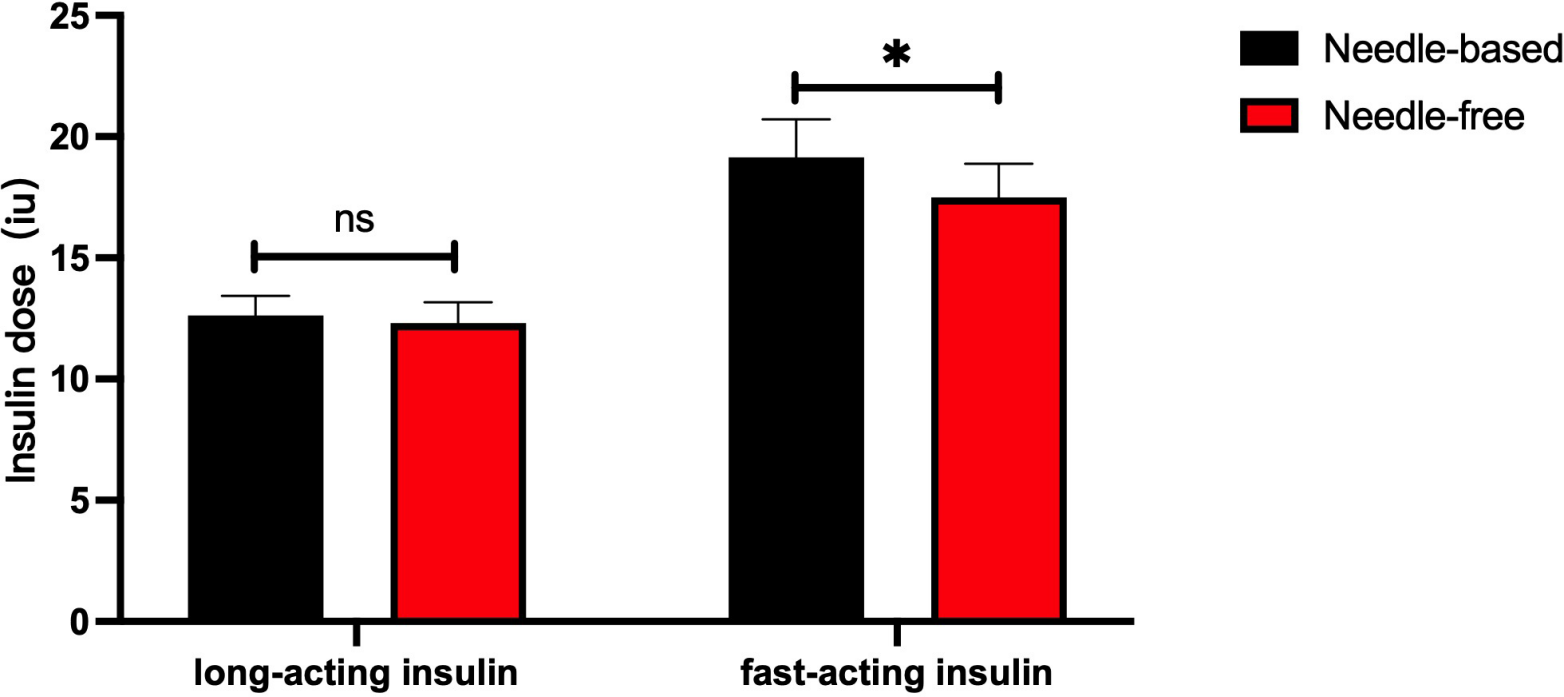
Comparison of the dose of long-acting and short-acting insulins after successively using the two injection devices. The abscissa shows the type of insulin (long-acting or short-acting), and the ordinate is the insulin injection dose (IU). The red box represents the insulin injection dose with a needle-free system, and the black box represents the insulin injection dose when using the needle system. The injection dose of long-acting insulin used by the two injection methods had no significant difference, p>0.05. The injection dose of quick-acting insulin when using the needle-free system was lower than that of the injection method requiring a needle, p<0.05. *p<0.05.

After intensive treatment with needle-free and needle injections, there was no significant difference in the dose of long-term insulin between the two groups (p > 0.05). However, the dose of fast-acting insulin decreased in patients using the needle-free system, when compared to those using the needle injection one (p < 0.05), as presented in **Figure 4**.

Patient satisfaction with the two kinds of injection devices was similar (P > 0.05), which were all above the general satisfaction level, as shown in **Table 2**. Of the 62 participants, 37 fulfilled the pain scale questionnaire after injecting insulin with the two types of injection devices. The pain degree of no needle injections was significantly lower than that of needle injections (p <0.05). Indeed, the rate of mild pain (67.6%) and moderate pain (64.9%) with needle injections (67.6% and 64.9%, respectively) was higher than that without needles (27% and 21.6%, respectively), as reported in **Table 3**. Patient pain perception of no-needle insulin injection was generally lower than when they used needle-injected insulin (p < 0.05).

**Table 2:** Comparison of satisfaction of patients with insulin injection between two methods.

	Needle-free group (N = 25)	Needle-based group (N = 37)	*t* value	*P* value
Degree of satisfaction (score, ± s)	7.29 ± 1.71	7.07 ± 1.39	-0.59	0.557

**Table 3:** Comparison of pain experience between two methods of insulin injection.

	Degree	Needle-free group (N = 37)	Needle-based group (N = 37)	*t* value	*P* value
Pain [case (%)]	Mild (1-3 points)	10 (27.0)	25 (67.6)	14.753	<0.001
	Moderate (4-6 points)	24 (64.9)	8 (21.6)		
	Severe (7-10 points)	3 (9.1)	4 (10.8)		

## Discussion

Here, a self-control study was carried out to compare the effects of a needle-free injection device with a needle injection device in the intensive treatment of T2DM patients. Results showed that glycemic control and fluctuation were improved during fasting and after breakfast when patients used a needle-free system, compared to those in use of needle injections. There was no significant difference in the long-acting insulin dose between the two injection methods, while patients needed a lower dose of short-acting insulin when using the needle-free injection system.

The results of this study are in part similar to those of previous reports on the conversion of needle injections to needle-free ones. Ji Qiuhe et al. (14) showed that needle-free injections could significantly reduce the dose of insulin glargine compared with needle systems, whereas the fasting blood glucose level was equivalent between the two groups. In parallel, Xie Xiaomin et al. showed (15) that the initial insulin injection dose needs to be reduced to avoid hypoglycemic events when replacing an insulin pen syringe with a needle-free syringe. However, we did not observe hypoglycemia in our study. Patients who received intensive treatment displayed improved fasting blood glucose control when using needle-free injections, despite using the same dose of long-acting insulin. Moreover, patients using needle-free injections needed a lower dose of fast-acting insulin. However, due to the small sample size and short observation time, the results of this investigation need to be confirmed by studies with large sample sizes.

The needle-free injection results in a good injection experience. Studies have shown that needle-free syringes produce very fine jets, and the diameter of the wound hole left on the skin surface is about 1/3 to 1/4 of that of insulin pens and traditional syringes (16). Therefore, the stimulation of nerve endings tends to be negligible, resulting in basically no pain. The results of our study showed that the pain sensation of needle-free injections was generally lower than that of needle ones. This is consistent with a previous study by Ji Linong et al. (17). There was no overall difference in satisfaction between the two injection systems.

Appropriate control of blood glucose in diabetic patients is the key to delaying the occurrence and development of diabetic complications. Long-term insulin needle injections can increase the mental and physical burden of diabetic patients. Furthermore, irregular insulin injection procedures can increase the incidence of bleeding, pain, subcutaneous fat hyperplasia, and increase the risk of cross-infection, and affect the therapeutic efficacy of patients (18, 19). Needle-free injection is a new method of insulin administration. The needleless injection is based on the principle of pressure jets. Through the pressure generated by the pressure device inside the needleless syringe, the liquid medicine instantly penetrates the human skin and reaches the subcutaneous tissue. The medicine liquid is then distributed under the skin, being absorbed more quickly and having a rapid action onset. Non-needle injections were shown to penetrate 4 to 6mm of skin, culminating in reduced pain and increased absorption area (9). Thus, the absorption rate of insulin is increased, the onset time of insulin is accelerated, and the peak time of the drug is shorter (20). As a result, in intensive treatment, the same dose of long-acting insulin using a needle-free delivery device can reach the peak drug concentration faster and reduce glucose levels more effectively. Similarly, to achieve the same blood glucose level, the dose of fast-acting insulin administered without a needle tends to be lower than that administered with a needle-based device. At the same time, needle-free injections can effectively reduce the adverse reactions caused by using a needle. It can relieve the patient’s psychological burden, reduce the patient’s fear of injection, reduce the occurrence of local complications, better control postprandial glucose, and improve patients’ compliance with insulin therapy and overall quality of life (21, 22).

The advantage of this study is that participants had their diet, exercise, sleep and other life aspects relatively stable during hospitalization. Thus, we were able to test the impact of the two drug administration methods without those confounders. However, this study also has limitations. For example, the sample size is small, and the observation time is short (although it exceeds the half-life of the drug) due to the influence of the hospital bed turnover rate. Thus, our findings need to be confirmed by a large sample size study. The injections, while given by a nurse, were not blinded to the patient due to the inherent limitations of the intervention and this is likely to have influenced their perception of pain. However, the glucose level can be influenced by many factors. The 7-point glucose values were regarded as the indicators of glucose control which might introduce a bias. Future studies which used continuous glucose monitoring system are still needed to confirm the results of this study.

In conclusion, needle-free injections can effectively reduce the occurrence of pain, and improve patient injection experience in T2DM patients undergoing in-hospital intensive glycemic control. Needle-free injections can be used in intensive insulin therapy, and the insulin dose does not need dose adjustment. The dose of fast-acting insulin may be decreased with the needle-free syringe which should be cautiously treated.

## Data availability statement

The original contributions presented in the study are included in the article/supplementary material. Further inquiries can be directed to the corresponding authors.

## Ethics statement

The studies involving human participants were reviewed and approved by Beijing Hospital Ethics Committe: 2020BJYYE c-026-01. The patients/participants provided their written informed consent to participate in this study. Written informed consent was obtained from the individual(s) for the publication of any potentially identifiable images or data included in this article.

## Author contributions

QW and LG conceived and designed the experiments. QW, WW, and QP wrote the manuscript and analyzed the data. WW and SY help to perform the statistical analysis. QW is the guarantor of this study and takes responsibility for the integrity of the data. All authors contributed to the interpretation of data and critical revision of the manuscript. QW has full access to all the data in the study and is the guarantor of this work and takes responsibility for the integrity of the data and the accuracy of the data analysis. All authors read and approved the final manuscript.
